# The Retrograde IFT Machinery of *C. elegans* Cilia: Two IFT Dynein Complexes?

**DOI:** 10.1371/journal.pone.0020995

**Published:** 2011-06-10

**Authors:** Limin Hao, Evgeni Efimenko, Peter Swoboda, Jonathan M. Scholey

**Affiliations:** 1 Department of Molecular and Cell Biology, University of California Davis, Davis, California, United States of America; 2 Department of Biosciences and Nutrition, Center for Biosciences at NOVUM, Karolinska Institute, Huddinge, Sweden; Inserm U869, France

## Abstract

We analyzed the relatively poorly understood IFT-dynein (class DYNC2)-driven retrograde IFT pathway in *C. elegans* cilia, which yielded results that are surprising in the context of current models of IFT. Assays of *C. elegans* dynein gene expression and intraflagellar transport (IFT) suggest that conventional IFT-dynein contains essential heavy (CHE-3), light-intermediate (XBX-1), plus three light polypeptide chains that participate in IFT, but no “essential” intermediate chain. IFT assays of XBX-1::YFP suggest that IFT-dynein is transported as cargo to the distal tip of the cilium by kinesin-2 motors, but independent of the IFT-particle/BBSome complexes. Finally, we were surprised to find that the subset of cilia present on the OLQ (outer labial quadrant) neurons assemble independently of conventional “CHE-3” IFT-dynein, implying that there is a second IFT-dynein acting in these cilia. We have found a novel gene encoding a dynein heavy chain, DHC-3, and two light chains, in OLQ neurons, which could constitute an IFT-dynein complex in OLQ neuronal cilia. Our results underscore several surprising features of retrograde IFT that require clarification.

## Introduction

Sensory cilia are cellular “antennae” that consist of a specialized ciliary membrane containing signaling molecules surrounding a microtubule-based axoneme, that project from the surface of many cells and detect environmental signals that control gene expression, cell behavior and development [1]. Sensory cilia are assembled by intraflagellar transport (IFT), a process discovered in *Chlamydomonas* involving the kinesin-2 driven anterograde movement of IFT particles from the base to the tip of the axoneme [2], [3], [4], [5], [6], [7], [8], [9], [10], [11]. IFT particles are multimeric protein complexes visible by EM as “trains” that are proposed to deliver assembly precursors, to the tip of the axoneme [12]. However, while progress has been made in studying the mechanism of anterograde IFT, the mechanism of retrograde IFT, which is proposed to recycle IFT components and turnover products back to the base of the cilium and which is known to be mediated by a form of dynein that we refer to here as IFT-dynein, remains relatively poorly understood.

Dyneins are large multi-subunit motor complexes that participate in ciliary assembly and beating, intracellular transport and mitosis [13]. Depending on the heavy chain sequence, cytoplasmic dyneins can be subdivided into the multifunctional cytoplasmic dynein 1 (containing the DYNC1 heavy chains plus intermediate, light intermediate, and several light chains) and the IFT-specific cytoplasmic dynein 2 (DYNC2) [14]. The retrograde motor for IFT was first discovered in sea urchin embryos as a form of cytoplasmic dynein whose expression is upregulated following de-ciliation, suggesting that it could be involved in cilia assembly [15]. Definitive evidence for a role of IFT-dynein in retrograde IFT was obtained with the demonstration that mutations in genes encoding the IFT-dynein heavy chains (named *che-3* in *C. elegans*) and light intermediate chains (*xbx-1* in *C. elegans*) produce short cilia that are filled with IFT particle subunits [16], [17], [18], [19], [20], [21]. Furthermore, retrograde, but not anterograde, IFT is disrupted in *che-3* mutants, which is consistent with the hypothesis that following loss of IFT-dynein function, IFT particles are transported only in the anterograde direction and thus accumulate at the tip of the axoneme [19]. However, much remains to be learned about the composition, mechanism, regulation and function of retrograde IFT and the IFT-dynein complex.

In order to improve our understanding of the subunit composition and organization of the IFT-dynein complex, we have performed an analysis of candidate dynein genes predicted to be present in the *C. elegans* genome. We found that, in addition to previously characterized IFT-dynein components, the expression of at least three dynein genes are associated with ciliated sensory neurons and participate in bidirectional IFT, but analysis of worms containing mutants in two of them revealed no structural or currently known functional defects in cilia. We obtained evidence that the IFT-dynein complex is carried as cargo by the concerted action of heterotrimeric and homodimeric kinesin-2 motors, but independent of the IFT particles and the BBSome (Bardet-Biedl syndrome protein complex). Finally, we identified a novel dynein heavy chain (DHC-3) that could potentially substitute for the conventional dynein heavy chain (CHE-3) in a subset of ciliated sensory neurons in this organism.

## Results

### Analysis of candidate dynein genes in post-embryonic *C. elegans*

Bioinformatic analysis reveals that the *C. elegans* genome encodes 17 candidate dynein genes [14], [22], [23]. The expression of a subset of these genes has been studied previously, revealing that the genes for IFT-dynein heavy chain, *che-3,* the light intermediate chain, *xbx-1,* and the TcTex-1-type dynein light chain, *dylt-2* (also called *xbx-2*), are expressed exclusively in ciliated sensory neurons [19], [20], [21], [24].

The 60 *C. elegans* ciliated sensory neurons are organized into several sensory organs called sensilla, of which the largest, named the amphids and phasmids, are located at the anterior and posterior ends of the animal, respectively. Most previous studies of IFT and ciliogenesis in *C. elegans* have focused on amphid and phasmid cilia. However, around the mouth, there also exist six outer labial sensilla (OL) and six inner labial sensilla (IL). Within the OL sensilla, there are two lateral symmetrical ciliated neurons called outer labial lateral neurons (OLL) plus four outer labial quadrant ciliated neurons (OLQ) [25], [26]. The ultrastructure of sensory cilia in these different sensilla has been examined in detail using electron microscopy [27], [28].

**Figure 1:**
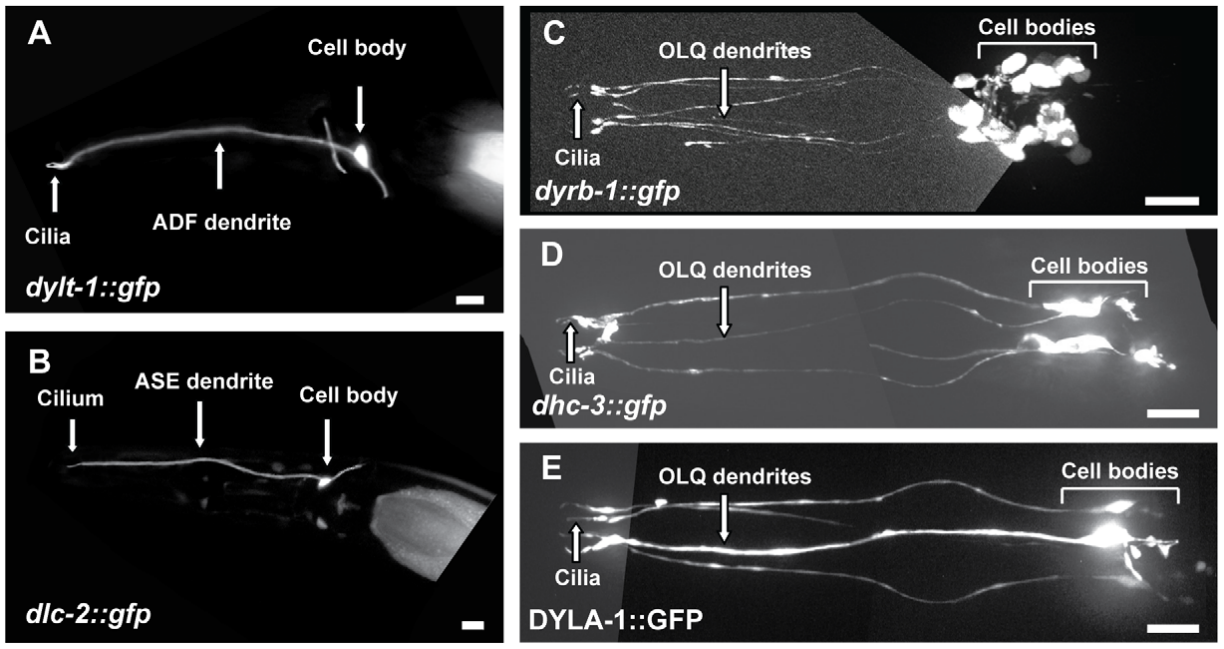
Expression patterns of candidate IFT-dynein genes. (A) A transcriptional *dylt-1::gfp* construct is expressed in the double-ciliated amphid neuron ADF. (B) A transcriptional *dlc-2::gfp* construct is expressed in the single-ciliated amphid neuron ASE. (C) A transcriptional *dyrb-1::gfp* construct is expressed in the ciliated neurons of the quadrant sensilla (OLQ neurons), and in other ciliated sensory neurons. (D) A transcriptional *dhc-3::gfp* construct is expressed in the OLQ neurons. (E) A translational DYLA-1::GFP construct is expressed in the OLQ neurons. In all the panels, Anterior is to the left and the horizontal bars represent 10 µm.

Here we used transcriptional GFP reporters to analyze the expression of the remaining dynein genes by constructing GFP fusions with the putative promoter regions of these genes and examining their expression patterns in transgenic worms (summarized in Table 1). We focused on those genes which, like the three previously studied IFT-dynein components, are expressed in ciliated sensory neurons, namely the Tctex-1-type dynein light chain, *dylt-1::GFP*, which contains an X-box motif in its promoter [21], [24] and is expressed in ADF (amphid chemosensory neuron F (double ciliated)) neurons in a DAF-19 transcription factor-dependent manner (Figure 1A); the dynein light chain, *dlc-2::GFP*, which is expressed in ASE (amphid chemosensory neuron E (single ciliated)) neurons (Figure 1B); the dynein roadblock-type light chain, *dyrb-1::GFP*, which is expressed in phasmid and OLQ neurons (Figure 1C); and finally two novel dynein-encoding genes, which are both expressed in QLQ neurons, namely a previously unreported dynein heavy chain gene, *dhc-3::GFP* (Figure 1D), and a dynein light chain gene with similarities to *Chlamydomonas* p28 axonemal dynein, DYLA-1::GFP (Figure 1E). The remaining genes analyzed are expressed in different types of cells, but their expression was not observed in ciliated sensory neurons (Table 1 and Figure S1).

**Table 1:** Post-embryonic expression patterns of *C. elegans* dynein genes.

Dynein subunit family	Gene name	Gene model	Available mutant alleles	Expression pattern	Reference
Dynein heavy chain	*dhc-1*	T21E12.4	*gk2765* and more	Pharynx, intestine, body wall muscles, hypodermis, head and tail neurons	McKay et al., 2003
	***che-3***	**F18C12.1**	*e1124, ok1574* and more	**Most or all CSN**	Signor et al., 1999; Wicks et al., 2000
	***dhc-3***	**B0365.7**	*ok5807*	**OLQ-type CSN,** HSN neurons, PVR, PVCR, PVWR tail neurons	Current work
Dynein intermediate chain	*dyci-1*	C17H12.1	*tm3700, tm4732*	Vulva muscles, body wall muscles, spermatheca	McKay et al., 2003
Dynein light intermediate chain	*dli-1*	C39E9.14	*js351, ku266, ku287*	Hypodermis, pharynx, unidentified neurons posterior to the nerve ring (non CSN)	Current work
	***xbx-1***	**F02D8.3**	*ok279*	Most or all CSN*	Schafer et al., 2003
Dynein light chain Tctex 1	***dylt-1***	**F13G3.4**	*ok416, ok417*	ADF-type CSN*, intestine	Current work
	***dylt-2***	**D1009.5**	*gk762, tm2097, tm2326*	Most or all CSN*	Efimenko et al., 2005
	*dylt-3*	T05C12.5	*tm2103*	Pharynx	Current work
Dynein light chain	*dlc-1*	T26A5.9	*tm3153*	Pharynx, intestine, body wall muscles, ventral nerve cord	McKay et al., 2003; Current work
	***dlc-2***	**M18.2**	None	**ASE-type CSN**, intestine, pharyngeal and head muscles	Current work
	*dlc-3*	Y10G11A.2	None	Body wall muscles, vulva muscles, distal tip cell	Current work
	*dlc-5*	Y73B6BL.43	None	Pharynx	Current work
	*dlc-6*	Y106G6G.3	None	No expression	Current work
Dynein light chain roadblock	***dyrb-1***	**T24H10.6**	*ok1931, tm2645*	**OLQ- and IL-type CSN (head), phasmid CSN (tail)**, intestine, unidentified neurons posterior to the nerve ring (non CSN)	Current work
Dynein light chain axonemal p28 like	***dyla-1***	**F41G4.1**	*tm3306*	**OLQ-type CSN**	Current work

Notes: Genes that are expressed in ciliated sensory neurons (CSN) are depicted in bold. * Gene expression is regulated by the DAF-19 transcription factor [24], [41]. Direct DAF-19 target genes contain the following X-box sequence motifs at specified distances upstream of the ATG: *xbx-1*GTTTCC AT GGTTAC (−93); *dylt-1* GTCTCC AT GACAAC (−1614); *dylt-2* GTTGCC AT GACAAC (−78).

### DYLT-1, DYLT-2 and DYRB-1 are components of the intraflagellar transport (IFT) machinery

**Figure 2:**
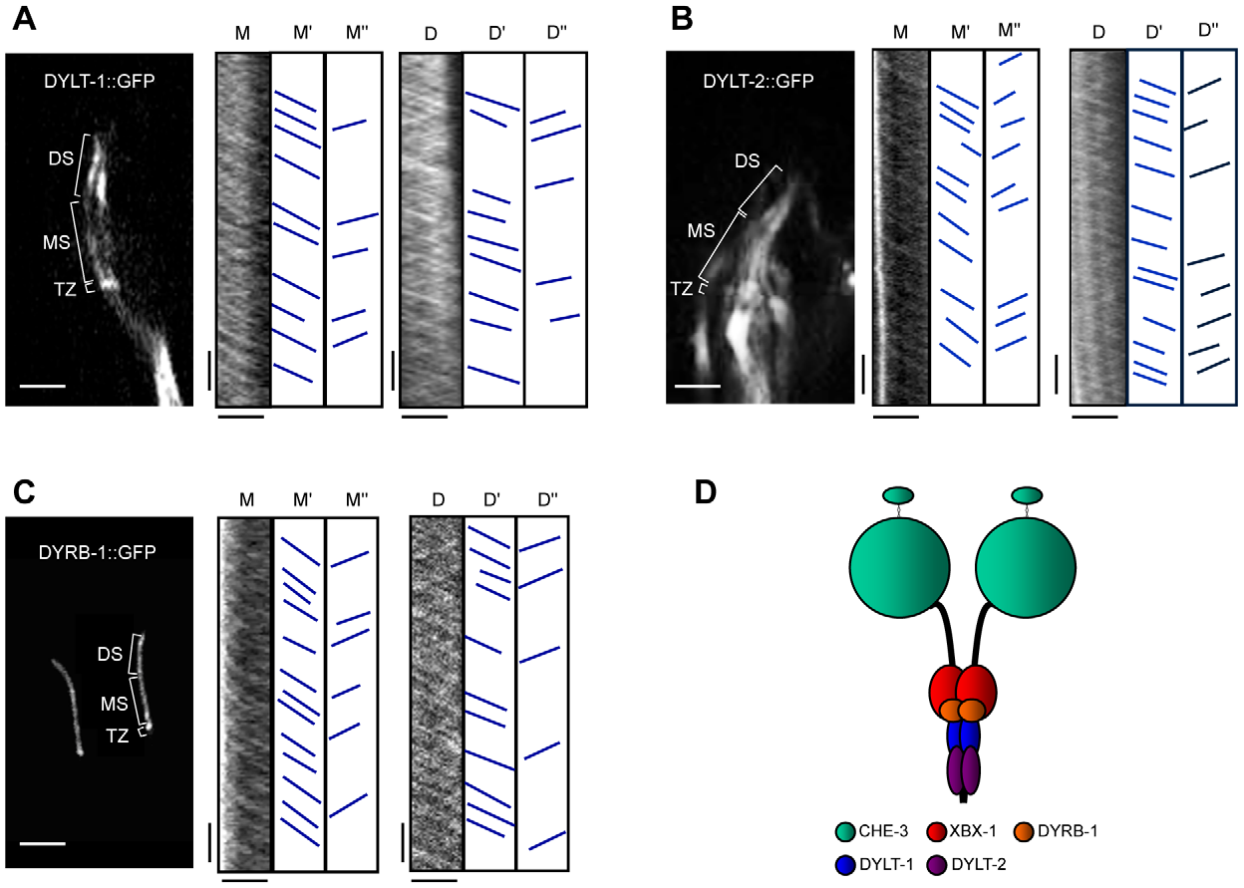
Three dynein light chains undergoing IFT indicate that they are components of the IFT-dynein complex. (A–C) IFT assays of three dynein light chains. The fluorescent micrographs of specific localization of DYLT-1::GFP in cilia of the ADF neuron (A), DYLT-2::GFP in cilia of amphid neurons (B) and DYRB-1::GFP in cilia of phasmid neurons (C) are shown in the left panels. Transition zones (TZ), middle (MS) and distal (DS) segments are indicated. Kymographs with corresponding cartoons indicate trajectories of moving particles along middle (M) and distal (D) segments in both anterograde (M', D') and retrograde (M'', D'') directions are shown in the middle and right panels. The horizontal bars represent 2.5 µm and the vertical bars represent 5 s. (D) A hypothetical model of the conventional IFT-dynein complex organization in *C. elegans*. The IFT-dynein complex in *C. elegans* consists of essential components, heavy chains (CHE-3) and light intermediate chains (XBX-1). The accessory components, Tctex 1-type dynein light chains such as DYLT-1 and DYLT-2, and a Roadblock-type light chain, DYRB-1, are non-essential and specifically associated with IFT-dynein in only subtypes of ciliated sensory neurons.

To investigate if the new putative IFT-dynein subunits participate in IFT, we generated transgenic worms expressing translational GFP fusions of DYLT-1, DYLT-2 and DYRB-1 and performed standard time-lapse fluorescence microscopy IFT assays on them [7], [9] (NB: We did not succeed in making similar transgenic animals expressing CHE-3 or DHC-3 translational GFP fusions; see Materials and Methods and Discussion). Using spinning disc confocal microscopy and kymography, in all three transgenic worm lines we observed robust diagonal tracks corresponding to fluorescent particles moving bi-directionally along the sensory cilia at the biphasic anterograde and unitary retrograde rates that are characteristic of IFT in this system (Figure 2, A–C, Table 2) [9]. This suggests that these three dynein subunits are novel components of the IFT machinery, and thus are likely to be components of the IFT-dynein complex (Figure 2D).

**Table 2:** IFT assays of IFT dynein light chain components.

Transgene	Middle segment velocity (µm/s)				Distal segment velocity (µm/s)			
	Anterograde IFT	N	Retrograde IFT	N	Anterograde IFT	N	Retrograde IFT	N
DYLT-1::GFP	0.75±0.13	156	−1.21±0.27	130	1.19±0.18	156	−1.23±0.22	109
DYLT-2::GFP	0.77±0.09	106	−1.13±0.24	84	1.25±0.21	205	−1.11±0.17	134
DYRB-1::GFP	0.79±0.08	181	−1.35±0.21	121	1.20±0.20	154	−1.30±0.18	126

Notes: N, particle number.

### Functional analyses of novel IFT-dynein components

**Figure 3:**
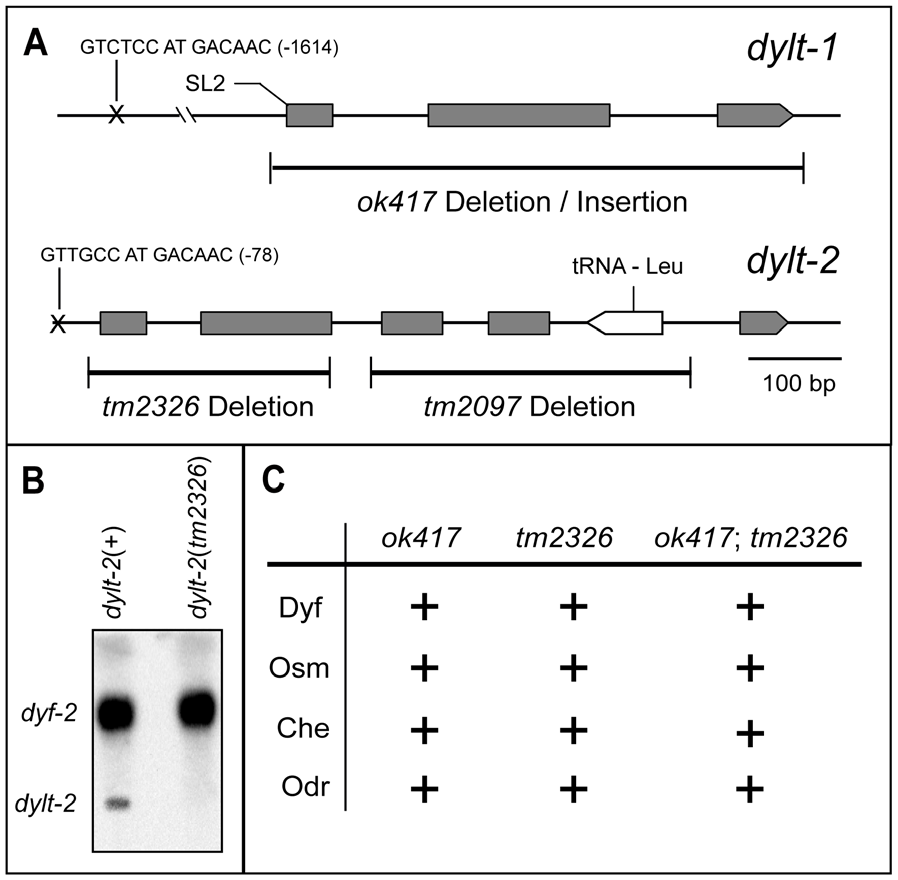
Functional analysis of the *dylt-1* and *dylt-2* genes. (A) Genomic organization of the *dylt-1* and *dylt-2* genes. The positions of X-box promoter motif sequences and the respective deletion mutant alleles are indicated. (B) Northern blot analysis of total RNA from *dylt-2*(+) and *dylt-2*(*tm2326*) mutant worms. In *dylt-2*(*tm2326*) mutants, expression of *dylt-2* was abolished in comparison with wild-type worms. The blot was also probed for *dyf-2* as a control. (C) Functional assays for *dylt-1* and *dylt-2* mutant worms. Both analyzed deletion mutants demonstrated wild-type responses (+) with regard to fluorescent dye filling (Dyf), avoidance of high osmotic strength (Osm), chemotaxis to sodium chloride (Che) and odorant sensation assays using Diacetyl (Odr). The same results were obtained with *dylt-1(ok417);dylt-2(tm2326)* double mutants.

To begin the functional investigation of the newly identified IFT-dynein components we focused our analyses on two genes, *dylt-1* and *dylt-2*, mutants of which were available. The *dylt-1(ok417)* deletion completely eliminates the coding sequence of the gene, thus representing a functional null allele (Figure 3A). The *dylt-2(tm2097)* deletion extends over 354 bp and eliminates a significant part of the Tctex-1 domain, as well as the entire D1009.t2 gene, which encodes a Leu-tRNA (Figure 3A). *dylt-2(tm2097)* mutant worms were homozygous lethal, possibly because of the D1009.t2 gene deletion. The other *dylt-2(tm2326)* deletion extends over 256 bp and eliminates the first 2 exons of the gene. Northern blot analysis demonstrated that the expression of *dylt-2* was abolished in *dylt-2(tm2326)* mutant worms (Figure 3B). *tm2326* thus most likely represents a functional null allele. Abnormalities of cilia structure or function can cause various mutant phenotypes in worms, including Dyf (fluorescent dye filling defective), Osm (avoidance of high osmotic strength defective), Che and Odr (chemo- and odorant sensation defective) [29], [30], [31]. Surprisingly, both analyzed deletion alleles demonstrated wild-type responses with regard to fluorescent dye filling, high osmotic strength avoidance, chemotaxis to sodium chloride and odorant sensation assays to diacetyl (Figure 3C). The same results were obtained with *dylt-1(ok417)*;*dylt-2(tm2326)* double mutants, suggesting that these IFT dynein subunits are not essential for ciliary structure and function, or that they are involved in very specific ciliary functions, which cannot be revealed by currently available assays.

### Anterograde transport of IFT-dynein complexes could be performed by two anterograde motors, independent of the IFT particles and the BBSome

**Figure 4:**
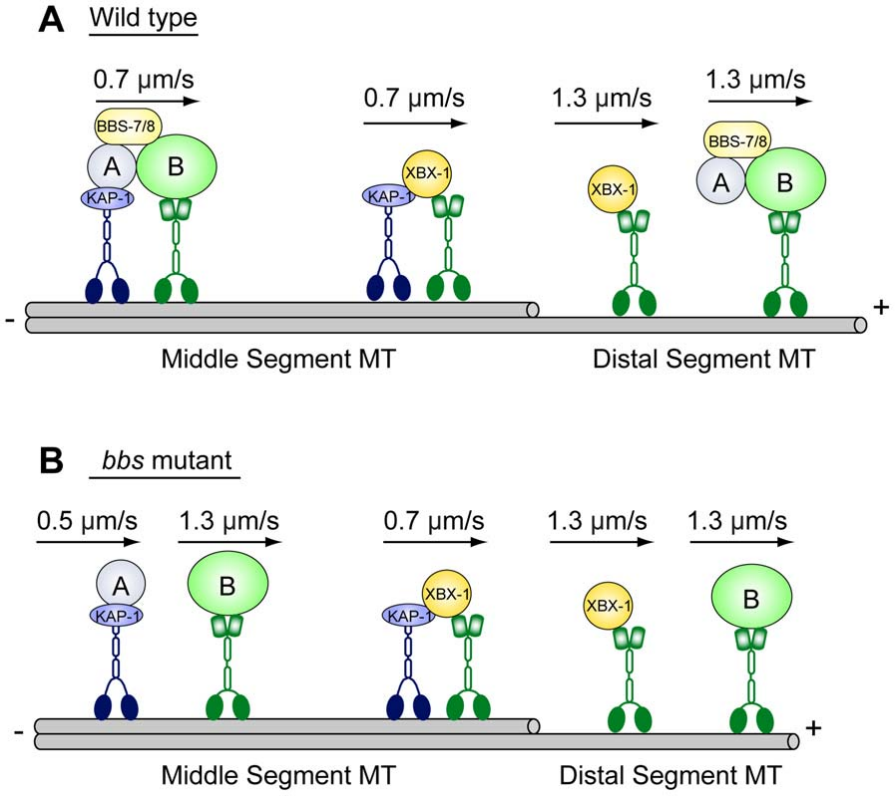
A model for XBX-1::YFP anterograde transport in *C. elegans* sensory cilia. (A) In wild type, XBX-1::YFP, likely associated with an IFT-dynein heavy chain, is transported separately from IFT particles by the coordinate action of the two IFT anterograde motors, kinesin-II and OSM-3, in the middle segment and by OSM-3 alone in the distal segment. (B) In *bbs* mutants, the transport of XBX-1::YFP is the same as in wild type, while the two IFT-A and IFT-B subcomplexes are separated and transported by kinesin-II and OSM-3, respectively.

We analyzed the anterograde transport of the IFT-dynein complex from the base to the tip of cilia using the tagged dynein light intermediate chain, XBX-1::YFP, and found rates that are consistent with the dynein being transported by the two IFT kinesins, heterotrimeric kinesin-II and homodimeric OSM-3 (Table 3). To further examine the transport dynamics we measured rates of anterograde transport of XBX-1::YFP in the *bbs* mutants *bbs-7 and -8,* in which IFT particles are proposed to break down into two subcomplexes, IFT-A, which is moved by kinesin-II alone at approximately 0.5 µm/s, and IFT-B, which is moved by OSM-3 alone at approximately 1.2 µm/s [32], [33]. Thus, we expected that if IFT-dynein were bound as cargo to a specific site on either IFT-A or IFT-B, XBX-1::YFP would behave like *every other* IFT-A and IFT-B marker that has been tested previously [34] and would move along the middle segments of the axoneme at rates characteristic of either kinesin-II or OSM-3, respectively. We were very surprised to find that XBX-1::YFP moved at the same rate in this mutant background as in wild-type animals, suggesting that it is carried by both anterograde motors acting together (Table 3). In support of this, loss of kinesin-II function in *bbs;klp-11* double mutants caused XBX-1::YFP to speed up and move at the faster rates characteristic of OSM-3 alone along the axoneme middle segments (Table 3). These data are not consistent with IFT-dynein being bound as cargo to the IFT particle complex (at least in a manner consistent with our current model for the organization of the IFT machinery in *C. elegans*) [32], [33], [34]. Instead, a plausible explanation for these results is that the IFT-dynein complex is carried as cargo that binds directly to the two anterograde motors, rather than being bound to the IFT particles, as proposed in Figure 4.

**Table 3:** XBX-1::YFP anterograde transport in different genetic backgrounds.

Genetic background	Middle segment		Distal segment	
	Anterograde IFT velocity (µm/s)	N	Anterograde IFT velocity(µm/s)	N
Wild type	0.77±0.08	105	1.30±0.18	122
*osm-3(p802)*	0.55±0.07	228	NA	NA
*klp-11(tm324)*	1.18±0.13	242	1.25±0.13	207
*bbs-7(n1606)*	0.73±0.09	124	1.24±0.17	94
*bbs-8(nx77)*	0.74±0.09	174	1.27±0.13	143
*bbs-7(n1606);klp-11(tm324)*	1.16±0.11	141	1.30±0.14	109
*bbs-8(nx77);klp-11(tm324)*	1.25±0.12	260	1.29±0.12	133

Notes: N, particle number. NA, not applicable.

### Ciliary structure in OLQ neurons is independent of conventional IFT-dyneins

**Figure 5:**
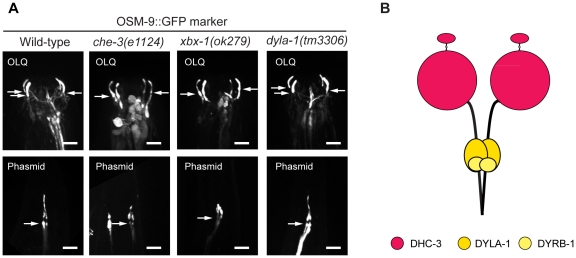
The gross structure of cilia associated with OLQ neurons is independent of conventional IFT-dynein. (A) A ciliary membrane marker, OSM-9::GFP, was used to visualize the structure of cilia of OLQ neurons and of phasmid neurons. Compared to those of wild type, the ciliary structure in mutants of two essential components of conventional dynein, *che-3(e1124)* and *xbx-1(ok279)*, is intact in the OLQ neurons, while truncated in the phasmid neurons. The ciliary structure is not grossly perturbed in the *dyla-1(tm3306)* mutant. The arrows point toward the ciliary base and the horizontal bars represent 5 µm in all panels. (B) A hypothetical new IFT-dynein could exist in *C. elegans* OLQ neurons.

We found that three dynein genes were expressed in OLQ neurons (Figure 1, C-E). One of them is a novel dynein heavy chain gene, *dhc-3*, whose protein product, DHC-3, could potentially represent a novel dynein complex. Thus we hypothesize that there may be two IFT-dyneins in the cilia of OLQ neurons, one based on CHE-3 (Figure 2D) and the other based on DHC-3 (Figure 5B), and to determine if both or only one of them are functional in OLQ cilia, we used an OSM-9::GFP marker to visualize both the OLQ ciliary structure and the phasmid ciliary structure. The assembly and maintenance of phasmid cilia is already known to require the conventional IFT dynein heavy chain, CHE-3 [19], [20]. As shown in Figure 5A, mutations in genes encoding two essential “conventional” IFT dynein subunits, CHE-3 and XBX-1, were associated with the formation of highly abnormal phasmid cilia (Figure 5A, lower panels). By contrast, in OLQ neurons, we were surprised to observe that cilia were indistinguishable from wild-type cilia in these mutant backgrounds (Figure 5A, upper panels), suggesting that conventional IFT-dynein is not essential for cilium biogenesis in OLQ neurons. We therefore hypothesize that the three candidate IFT genes expressed in OLQ neurons, namely *dhc-3*, *dyrb-1* and *dyla-1* could form a novel IFT-dynein that is required to form the cilia in OLQ neurons (Figure 5B). A mutation in one of these subunits, *dyla-1(tm3306*) was not associated with ciliary defects in OLQ cilia (Figure 5A), indicating that this subunit of the hypothesized second IFT-dynein is not an essential component of retrograde IFT in these cilia. DHC-3 might be an essential component of this putative IFT-dynein. Further testing of this hypothesis will require loss-of-function mutants in the gene *dhc-3*. Although a mutation in the gene *dhc-3* was recently isolated (*ok5807*; Table 1), this allele is merely a point mutation residing within an intron: *ok5807* is thus unlikely to affect protein expression or function.

## Discussion

The nematode *C. elegans* represents an attractive model to study IFT-dynein and retrograde IFT in sensory cilia of ciliated neurons. The *C. elegans* genome encodes homologs of both cytoplasmic dynein and IFT-dynein as well as important components of the IFT machinery, but no axonemal dyneins, allowing dissection of dynein molecules specifically required for IFT in a system lacking axonemal dyneins. In *C. elegans* the *che-3* gene encodes the IFT dynein heavy chain [19], [20] and the *xbx-1* and *dylt-2* (also called *xbx-2*) genes encode IFT-dynein light intermediate and Tctex-1-type light chains, respectively [21], [24]. In the current study, our post-embryonic expression analysis of dynein genes further demonstrated that more members of the dynein gene class, *dylt-1, dlc-2*, *dyrb-1* and *dyla-1*, encoding different dynein light chains, as well as the gene *dhc-3*, encoding a novel dynein heavy chain, are expressed in ciliated sensory neurons and that *dylt-1, dylt-2 and dyrb-1* participate in IFT. We further continued to analyze available mutants of novel IFT-dynein genes. However, our functional analyses of *dylt-1* and *dylt-2* mutant worms revealed no structural or functional abnormalities in cilia, suggesting that these light chains are not essential and may be responsible for binding very specific, hitherto unidentified cargoes.

The data obtained in our study allowed us to propose a model for the “conventional” IFT-dynein complex in *C. elegans* (Figure 2D). According to this model, this complex consists of heavy (CHE-3), light intermediate (XBX-1), Tctex-1-type light (DYLT-1 and DYLT-2) and Roadblock light (DYRB-1) chains. While CHE-3, XBX-1 and DYLT-2 are expressed in most or all ciliated sensory neurons and comprise the core of the IFT-dynein complex, DYLT-1 and DYRB-1 are specifically associated with IFT-dynein in only a subset of ciliated sensory neurons. Further experimental approaches, for example motility assays of novel dynein components, the expression of translational GFP constructs in *bbs* and in two different IFT (subcomplex A and B) mutant backgrounds, as well as expression of other IFT proteins in dynein mutant backgrounds will allow researchers to establish the complete molecular composition of the IFT-dynein complex and understand its role in cilia biogenesis. A notable result is the apparent absence of an intermediate chain (DYCI-1) [35] that is required for “conventional” IFT-dynein to function. This contrasts with other organisms such as *Chlamydomonas*, where a *bona fide* intermediate chain has been identified as an important component of IFT-dynein [36].

One surprising result of our dissection of the retrograde IFT transport machinery of *C. elegans* sensory cilia is the finding that the IFT-dynein complex is transported differently from IFT subcomplex A and B subunits, as monitored by IFT assays using a YFP-tagged XBX-1 subunit. We had assumed that IFT-dynein would be transported as cargo from the base to the tip of the axoneme as a result of being bound to either IFT-A or IFT-B subcomplexes, which are transported separately by kinesin-II and OSM-3 in *bbs* mutants, as assayed using multiple components of the IFT particles [32], [33], [34]. Unexpectedly, however, unlike all IFT-A and IFT-B-associated proteins analyzed so far, the rates of anterograde transport of IFT-dynein were not affected by the loss of BBS subunits, leading us to hypothesize that IFT-dynein is transported by kinesin-II and OSM-3 in a manner that is independent of the IFT-particles and the BBSome, in a separate pathway as indicated in Figure 4. However, while consistent with the available data, this hypothesis is speculative and requires further testing. For example, the model predicts that IFT-dynein could bind directly to the kinesin-2 motors, which in principle could be tested in biochemical binding assays, but such assays of IFT motor-cargo interactions are notoriously difficult [37] and lie beyond the scope of the current study.

Potentially the most interesting finding in our current study was obtained as a result of observing the expression patterns of two presumptive IFT-dynein heavy chains, CHE-3 and DHC-3, and examining the structure of cilia in OLQ neurons containing mutations in the “conventional” IFT dynein (i.e. comprising the CHE-3 heavy chain). Based on the results obtained we propose that the DHC-3 dynein heavy chain, together with DYLA-1 and DYRB-1 could form a second IFT-dynein complex (Figure 5) that functions specifically in OLQ neurons to build these cilia. This hypothesis needs further testing through the acquisition of loss-of-function mutants in the *dhc-3* gene and observing whether fluorescently tagged DHC-3 protein moves along OLQ neuronal cilia in direct assays of IFT. These are both likely to be long-term projects, however. Indeed, we have already placed much effort into the creation of transgenic worms expressing fluorescently tagged CHE-3 dynein heavy chain, but for technical reasons this has proven unsuccessful so far, likely because of the extremely large size of dynein heavy chains.

In summary, our analysis of IFT-dynein and retrograde IFT in *C. elegans* cilia has uncovered several surprising results which we hope and anticipate will pave a way to a clearer understanding of the little-known molecular mechanism of this transport pathway. It has yielded information that should focus attention on several specific aspects of retrograde IFT that require detailed examination in the future.

## Materials and Methods

### Worm strains

Growth and culture of *C. elegans* strains were carried out following standard procedures [38]. The strains that were used for this study can be found in the Table S1. All strains used and strain construction details are available on request.

### Generation and analysis of GFP expression constructs and transformation

Transcriptional GFP expression constructs were designed by inserting promoter regions and the first several codons of a gene of interest into the GFP expression vectors pPD95.75 or pPD95.77 (gift from A. Fire). Translational GFP expression constructs were obtained by cloning the respective ORFs of a gene of interest into appropriate sites of previously designed transcriptional constructs. All constructs were verified by sequencing for correct translational reading frames. Preparing translational GFP expression constructs with the two dynein heavy chain genes (*che-3* and *dhc-3*) was not successful using a conventional PCR-cloning approach due to the large size of both genes (>12 kb). A trial using a novel recombination strategy [39] allowed us to obtain a DHC-3::dsRed translational fusion. However, the transgenic worms carrying this construct did not show any fluorescence signal. Likewise, the transgenic worms carrying DLC-2::GFP gave rise to extremely low fluorescence signal in 2–3 cells in the head, which also made it impossible to analyze further. Transgenesis: Adult hermaphrodites were transformed using standard protocols [40]. Constructs were injected typically at 10–100 ng/µl along with the coinjection marker pRF4 (containing the dominant marker *rol-6(su1006)*). JT6924 and JT204 worm strains were used to test transcriptional constructs for GFP expression and dependence on DAF-19 transcription factor function [24], [41]. The worm strain JT6924 *daf-19(m86); daf-12(sa204)* was used as a *daf-19* mutant background. Worms of this genotype exhibit a Daf-d (dauer larva formation - defective) phenotype and do not require the recovery of dauers. In this case, JT204 *daf-12(sa204)* worms were used as a wild-type background with regard to *daf-19*.

### Microscopy and imaging

GFP expression patterns were analyzed in stable transgenic lines at 1000x magnification by conventional fluorescence microscopy (Zeiss Axioplan 2) or confocal microscopy (Olympus). Expression patterns were examined in at least two independent transgenic lines. Determination of neuronal cell anatomies and identities followed published descriptions [26], [42]. Intraflagellar transport (IFT) was assayed as previously described [7], [9], [32], [43]. Transgenic worms anesthetized with 10 mM levamisole were mounted on agar pads and maintained at 21°C. Images were collected on an Olympus microscope equipped with a 100x, 1.35 NA objective and an Ultraview spinning disc confocal head at 0.3 second/frame for 2–3 minutes. Kymographs and movies were created using Metamorph (Molecular Devices) software.

### Genetic characterization of dynein mutants

All deletion alleles analyzed in this study were generated by the *C. elegans* Gene Knockout Consortium (http://celeganskoconsortium.omrf.org) or by the National BioResource Project in Japan (http://shigen.lab.nig.ac.jp/c.elegans) using publicly available methodology. The original mutant strains RB663 *dylt-1(ok417)* I and FX02326 *dylt-2(tm2326)* X were outcrossed three times with wild type N2 and the following worm strains: CB2167 *dpy-5(e61) unc-13(e1091)* I and MT3324 *lon-2(e678) egl-15(n484)* X, respectively. Outcrossed worms resulted in homozygous mutant strains containing the respective mutant alleles, OE3246 and OE3317. These strains were then used as the basis for further analysis.

### Northern blot analysis

Total RNA for Northern blot analysis was isolated from mixed stage worms by addition of TRIZOL reagent (Invitrogen) and further purified with an RNeasy kit (Qiagen). *dyf-2* [44] and *dylt-2* probes were labeled with a chemiluminescent reagent using the AlkPhos Direct labeling and detection kit (Amersham Biosciences).

### Fluorescent dye-filling and behavioral assays

Fluorescent dye-filling assays were performed as described [31] using the fluorescent dyes DiI (1,1′-Dioctadecyl-3,3,3′,3′-tetramethylindocarbocyanine iodide) and FITC (5(6)-fluorescein isothiocyanate). Stained adult hermaphrodites were analyzed at 1000x magnification by conventional fluorescence microscopy (Zeiss Axioplan 2). The fluorescent dye-filling defective worm strain CB3323 *che-13(e1805)* was used as a control for the Dyf phenotype. Osmotic avoidance assays were performed essentially as described [30] by testing the ability of adult hermaphrodite worms to cross a ring of high osmotic strength (8 M glycerol). All tests were done during a time period of 10 min. The osmotic avoidance defective worm strain CB3323 *che-13(e1805)* was used as a control for the Osm phenotype. Population chemotaxis to water-soluble chemicals assays were performed as described [20] using 50 mM sodium chloride. The chemotaxis defective worm strain CB1033 *che-2(e1033)* was used as a control for the Che phenotype. Population chemotaxis to odorants assays were performed as described [29] using diacetyl (10^−3^ dilution). The chemotaxis defective worm strain CB1033 *che-2(e1033)* was used as a control for the Odr phenotype.

## Supporting Information

Figure S1 **Expression patterns of dynein genes that are not expressed in ciliated sensory neurons.** (A) A transcriptional *dli-1::gfp* construct is expressed mostly in hypodermal cells. Expression was also observed in the pharynx and in some non-ciliated neurons posterior to the nerve ring (data not shown). (B) A transcriptional *dylt-3::gfp* construct is expressed in pharyngeal muscles. (C) A transcriptional *dlc-1::gfp* construct is expressed in many different types of cells, including pharynx, intestine, and body wall muscles. Expression was also observed in the ventral nerve cord (data not shown). (D) A transcriptional *dlc-3::gfp* construct is expressed in body wall muscles and vulva muscles. Expression was also observed in the distal tip cell (data not shown). (E) A transcriptional *dhc-3::gfp* construct is expressed in the HSN neurons. (F) A transcriptional *dhc-3::gfp* construct is expressed in three neurons in the tail, which are likely PVR, PVCR, PVWR. (G) A translational DLC-5::GFP construct is expressed in the pharynx. The horizontal bars represent 10 µm in panels A, B, E–G, and 50 µm in panels C and D.
(TIF)

Table S1 **Worm strains used in our study.**
(DOCX)
