# Supplementary figures and images for: The Retrograde IFT Machinery of C. elegans Cilia: Two IFT Dynein Complexes?
Source: PLoS One. 2011 Jun 10;6(6):e20995. doi: 10.1371/journal.pone.0020995 (PMC3112216; doi:10.1371/journal.pone.0020995)

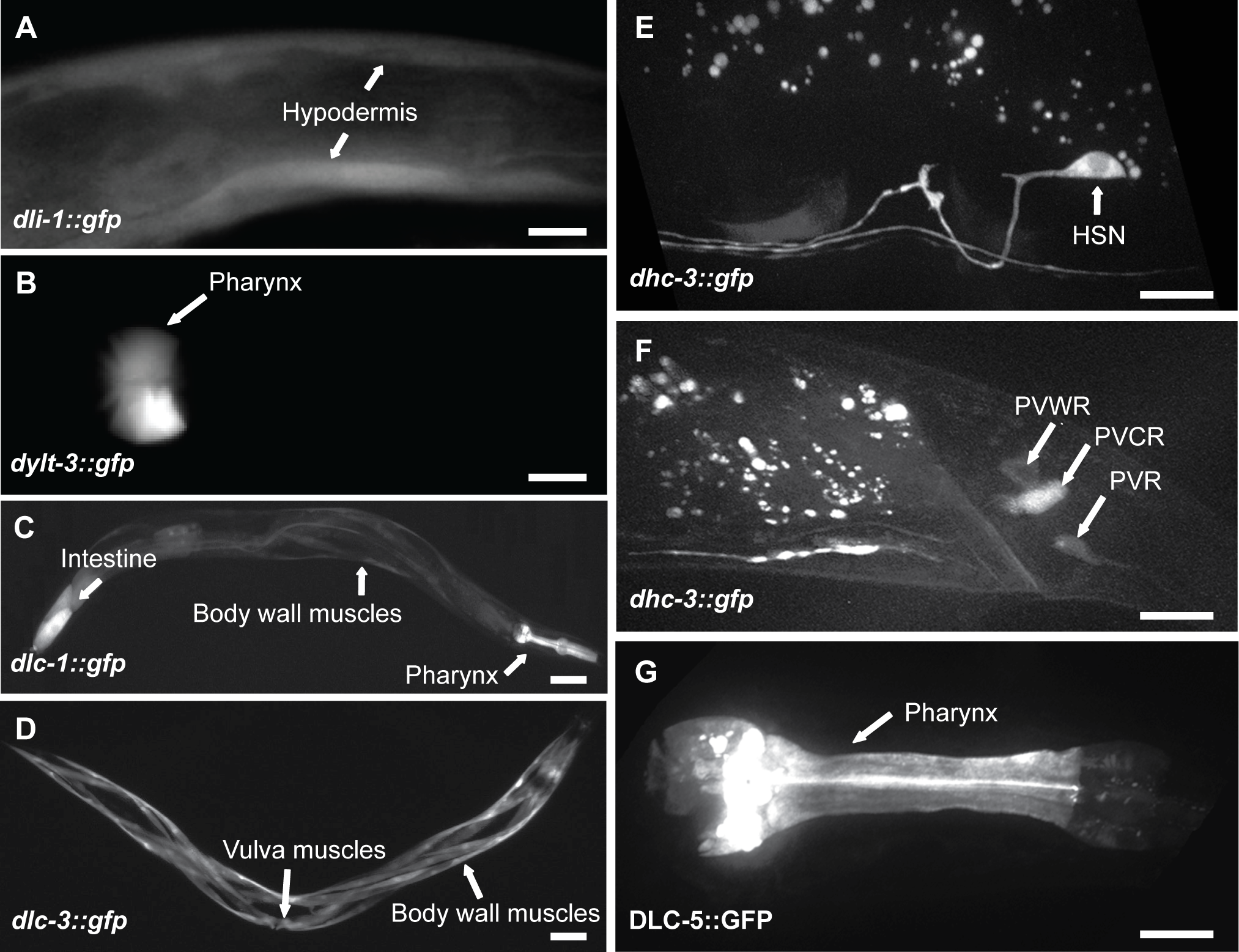

Supplement: Figure S1 — Expression patterns of dynein genes that are not expressed in ciliated sensory neurons. (A) A transcriptional dli-1::gfp construct is expressed mostly in hypodermal cells. Expression was also observed in the pharynx and in some non-ciliated neurons posterior to the nerve ring (data not shown). (B) A transcriptional dylt-3::gfp construct is expressed in pharyngeal muscles. (C) A transcriptional dlc-1::gfp construct is expressed in many different types of cells, including pharynx, intestine, and body wall muscles. Expression was also observed in the ventral nerve cord (data not shown). (D) A transcriptional dlc-3::gfp construct is expressed in body wall muscles and vulva muscles. Expression was also observed in the distal tip cell (data not shown). (E) A transcriptional dhc-3::gfp construct is expressed in the HSN neurons. (F) A transcriptional dhc-3::gfp construct is expressed in three neurons in the tail, which are likely PVR, PVCR, PVWR. (G) A translational DLC-5::GFP construct is expressed in the pharynx. The horizontal bars represent 10 µm in panels A, B, E–G, and 50 µm in panels C and D. (TIF) [file pone.0020995.s001.tif]
