# Supplementary material for: The Retrograde IFT Machinery of C. elegans Cilia: Two IFT Dynein Complexes?
Source: PLoS One. 2011 Jun 10;6(6):e20995. doi: 10.1371/journal.pone.0020995 (PMC3112216; doi:10.1371/journal.pone.0020995)
Supplement: Table S1 — Worm strains used in our study. (DOCX) [file pone.0020995.s002.docx]

**Table S1.** Worm strains used in our study.

| **Strain name** | **Genotype** |
| --- | --- |
| N2 | Wild type |
| CB1033 | *che-2*(*e1033*) |
| CB2167 | *dpy-5(e61)* *unc-13(e1091)* |
| CB3323 | *che-13(e1805)* |
| FX02326 | *dylt-2(tm2326)* |
| JT204 | *daf-12(sa204)* |
| JT6924 | *daf-19(m86); daf-12(sa204)* |
| MT3324 | *lon-2(e678) egl-15(n484)* |
| OE3246 | *dylt-2(tm2326)* |
| OE3275 | *daf-12(sa204)*; *ofEx207* [*dylt-1::gfp; rol-6(su1006)*] |
| OE3277 | *daf-19(m86)*; *daf-12(sa204)*; *ofEx209* [*dylt-1::gfp*; *rol-6(su1006)*] |
| OE3279 | *daf-12(sa204)*; *ofEx211* [*dylt-3::gfp*; *rol-6(su1006)*] |
| OE3314 | *dylt-1(ok417)*; *ofEx238* [*dylt-1::gfp*; *rol-6(su1006)*] |
| OE3317 | *dylt-1(ok417)* |
| OE3493 | *dylt-1(ok417)*; *dylt-2(tm2326)* |
| OE3650 | *daf-12(sa204)*; *ofEx450* [*dlc-1::gfp*; *rol-6(su1006)*] |
| OE3653 | *daf-12(sa204)*; *ofEx453* [*dlc-2::gfp*; *rol-6(su1006)*] |
| OE3654 | *daf-12(sa204)*; *ofEx454*[*dlc-3::gfp*; *rol-6(su1006)*] |
| OE3655 | *daf-12(sa204)*; *ofEx455* [*dyrb-1::gfp*; *rol-6(su1006)*] |
| OE3658 | *ofEx458*[*dli-1::gfp; elt-2::mcherry*] |
| RB663 | *dylt-1(ok417)* |
| LH93 | *bbs-7(n1606); Ex*[*XBX-1::YFP*; *rol-6(su1006)*] |
| LH95 | *bbs-8(nx77); Ex*[*XBX-1::YFP*; *rol-6(su1006)*] |
| LH91 | *osm-3(p802); Ex*[*XBX-1::YFP*; *rol-6(su1006)*] |
| LH92 | *klp-11(tm324); Ex*[*xbx-1::YFP*; *rol-6(su1006)*] |
| LH96 | *bbs-7(n1606);klp-11(tm324); Ex*[*xbx-1::YFP; rol-6(su1006)*] |
| LH97 | *bbs-8(nx77);klp-11(tm324); Ex*[*xbx-1::YFP; rol-6(su1006)*] |
| LH232 | *Ex232*[*DYLA-1::GFP*; *rol-6(su1006)*] |
| LH239 | *Ex239*[*dhc-3:gfp*; *rol-6(su1006)*] |
| LH252 | *Ex252*[*dlc-5::gfp*; *rol-6(su1006)*] |
| LH255 | *Ex255*[*dlc-6::gfp*; *rol-6(su1006)*] |
| LH266 | *dyla-1(tm3306); Ex*[*OSM-9::GFP*] |
| LH267 | *xbx-1(ok279); Ex*[*OSM-9::GFP*] |
| LH206 | *che-3(e1124); Ex*[*OSM-9::GFP*] |
